# Cardiovascular risk factors and modern therapeutic strategies in children and adolescents with type 1 diabetes to prevent future diabetic angiopathy in the era of innovative miRNAs biomarkers

**DOI:** 10.3389/fendo.2025.1638770

**Published:** 2025-09-08

**Authors:** Joanna Peczyńska, Emilia Odyjewska, Kamila Koszykowska, Milena Jamiołkowska-Sztabkowska, Artur Bossowski, Barbara Głowińska-Olszewska

**Affiliations:** Department of Pediatrics, Endocrinology, and Diabetology with Cardiology Division, Medical University of Bialystok, Białystok, Poland

**Keywords:** type 1 diabetes, children, cardiovascular risk factors, cardiovascular complications, miRNA, precise medicine

## Abstract

Type 1 diabetes (T1D) in children is a serious, chronic, incurable disease associated with the frequent and early occurrence of additional, well-known cardiovascular risk factors and exacerbation of the risk of future cardiovascular diseases (CVD). Lately, accumulating evidence suggests that exosomal miRNAs play a major role not only in the pathophysiology of T1D but also in its late complications. Since premature CVD is one of the leading causes of morbidity and mortality in diabetes, considerable efforts have been made to define the molecular and pathological features and to develop new diagnostic and therapeutic strategies. Dysregulation of the expression or function of various miRNAs may affect angiogenesis, vascular inflammation, or cardiac remodeling, which play key roles in the development and progression of cardiovascular complications. While CVD usually appear in adulthood, pathology and early markers may appear during adolescence, emphasizing the need for careful monitoring and prevention in this age group. In this narrative review, we aimed to summarize the latest findings on miRNAs and their role as biomarkers of cardiovascular risk factors and subsequent complications in children with T1D, presenting promising candidates for clinical applications.

## 1 Introduction

Type 1 diabetes (T1D) is a chronic autoimmune disease, increasingly common in the pediatric population, characterized by a progressive destruction of insulin-secreting β-cells of the pancreas (1, 2). As a result of loss of their function, patients are completely dependent on exogenously administered insulin to maintain normal blood glucose levels. It is common knowledge that long-term excessively high glucose concentrations eventually lead to various complications such as cardiovascular disease, diabetic retinopathy, neuropathy, renal failure, hearing impairment or more (3). Therefore, it is important to maintain glycemic control, appropriate for children and adolescents, within the recommended limits of glycated hemoglobin (HbA1C) <7% (<53 mmol/mol) and to spend more than 70% of the time in range (TIR) (70 – 180 mg/dL [3.9 – 10.0 mmol/L]) as measured by continuous glucose monitoring (CGM) to lower the hazard and incidence of macrovascular and microvascular complications (4). For selected individuals, an even more stringent HbA1C target of <6.5% (<48 mmol/mol) is suggested. Although the above principles help to reduce the risk or delay the occurrence of later diabetic macrovascular complications, unfortunately they cannot always be prevented. Diabetic angiopathy refers to a group of vascular diseases that develop as a consequence of diabetes and include damage to blood vessels, both small (microangiopathy) and large (macroangiopathy), caused by high blood sugar levels. We can distinguish an early, preclinical phase, in which patients often exhibit no clinical symptoms, followed by the development of measurable retinal, renal, or nerve damage, under the influence of persistent hyperglycemia, as well as endothelial dysfunction (ED), thickening of the carotid intima media, and atherosclerosis, ultimately associated with an increased risk of heart attack, stroke, or lower limb amputation. Childhood T1D is associated with an increased incidence of additional risk factors already at a young age, such as excess body weight, further aggravated by insulin therapy, lipid profile disorders or hypertension, which, cumulatively, may lead to a type of metabolic syndrome (5). In addition, early vascular changes in the form of endothelial dysfunction, arterial stiffness or increased carotid intima-media thickness (cIMT) have been demonstrated in children with type 1 diabetes (6, 7). All of the above-mentioned causes may contribute to further macrovascular complications, including peripheral artery disease (PAD), coronary artery disease (CAD) leading to myocardial infarction or cerebrovascular disease. Currently, known risk factors and previously studied biomarkers do not fully explain the pathophysiology, frequency of vascular changes, and reduced life expectancy in type 1 diabetes. Knowledge of the molecular mechanisms underlying the pathogenesis of diabetic micro- and macroangiopathy is extremely important for prevention, delaying the onset and, in particular, identifying new targets for the treatment of cardiovascular complications of diabetes. In the pursuit of precision medicine, new biomarkers are sought, which emerge to be miRNAs.

**Figure 1:**
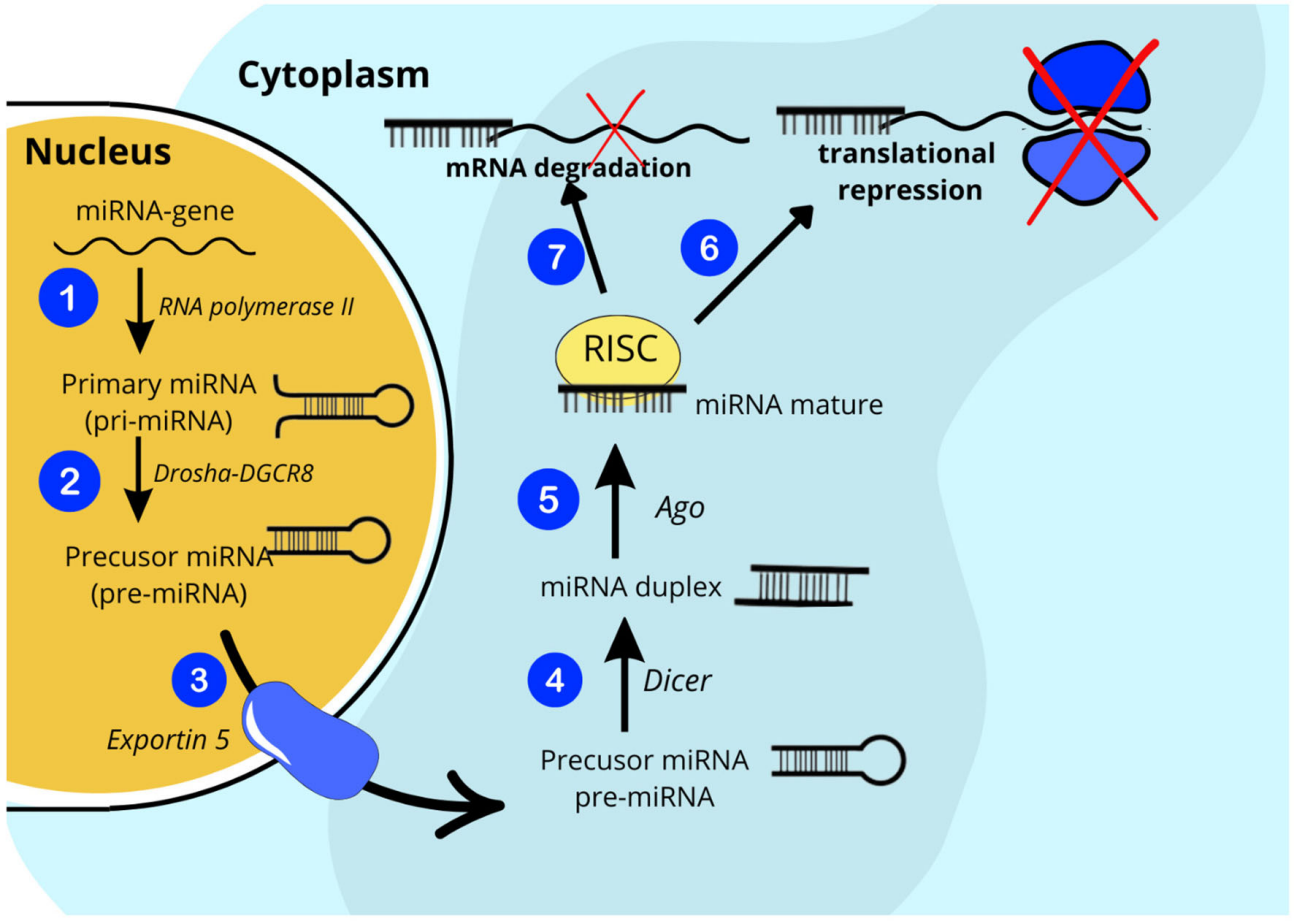
Simplified scheme of miRNA production and its role in the body.

Recently, accumulating evidence suggests that exosomal miRNA plays a tremendous role not only in the pathophysiology of T1D but also in its late complications (8, 9). Exosomes are nanometer-sized bilayer extracellular vesicles (EVs) (30 – 200 nm in diameter) that can be secreted by virtually all cell types in response to internal or external stimuli (10). Once secreted, EVs gain access to the interstitial space and ultimately to the circulation, where they exert local paracrine or distal systemic effects. The number and molecular cargo of exosomes are tightly regulated by physiological and pathological factors. For these reasons, EVs are important components involved in intercellular and interorgan communication via their contents, such as proteins, lipids, and RNAs, including microRNAs, which are attributed to the greatest share of biological effects of exosomes (11). MiRNAs are a class of small (~ 22 nucleotides in length) single-stranded non-coding RNAs that act as regulators of protein-coding genes. By binding to target messenger ribonucleid acids (mRNAs), they have the ability to modulate gene expression at the posttranscriptional level by facilitating sequence-specific RNA interference, either by inhibiting translation or by promoting mRNA degradation (**Figure 1**) (12, 13). To date, more than 2,600 different mature human miRNAs have been reported in the miRBase database (14). Surprisingly, together they may have the capacity to regulate even about 30% of all genes in the human genome. Bioinformatic predictions indicate that one miRNA can influence changes in more than a hundred mRNAs and that a single mRNA can be regulated by many different miRNAs. It has been presented that miRNA expression levels are tissue-specific, stable in body fluids even long after sample collection and freeze-thaw cycles, and can be collected minimally invasively from readily accessible biofluids, making miRNAs promising candidates for biomarkers enabling accurate diagnosis and prognosis of the progression of a wide range of diseases (15–18). On the other hand, the reproducibility of miRNA biomarker assays is often limited due to the combined influence of biological and technical factors, including limited understanding of the normal range of miRNA expression in different body fluids and the lack of disease specificity of some miRNAs, inconsistent sample preparation, which may affect molecular stability or measurement techniques, or the lack of standardized data normalization across studies, which makes it difficult to translate research results into clinical practice (19).

Cardiovascular disease (CVD) is considered to be one of the leading causes of morbidity and mortality in young and older adults with type 1 diabetes and is more common in this population compared to healthy individuals (20), which is why extraordinary efforts have been made to define the molecular and pathological features of the diseased heart and vasculature as well as to develop novel diagnostic and therapeutic strategies. MiRNAs have been associated with nearly all cardiovascular diseases in which they have been studied, including heart failure, cardiac hypertrophy, post-myocardial infarction remodeling, arrhythmia, atherosclerosis, atrial fibrillation, and peripheral artery disease. Dysregulation of the expression or function of various miRNAs may affect angiogenesis, vascular inflammation, or cardiac remodeling, which play a key role in the development and progression of cardiovascular complications (21).

We would like to emphasize that the use of miRNA determination could become a routine approach in the prognosis, early diagnosis and monitoring the progression of cardiovascular complications and would also provide the development of better therapeutic strategies, implementing precision medicine conception. In the following review, we aimed to discuss the latest findings regarding miRNAs and their possible role as biomarkers in cardiovascular complications in children and adolescents with type 1 diabetes mellitus. We have decided to focus on highlighting the most relevant miRNAs that have been described in many studies and seem promising for clinical applications in this specific group of patients. The miRNAs described in this article and their expression in individual risk factors and complications are summarized in **Table 1A** (risk factors) and **Table 1B** (complications).

**Table 1A:** The miRNAs described in the article and their expression in individual risk factors.

Risk factor	Expression	miRNA		
**Type 1 diabetes**	**Elevated**	miR-144-5p **miR-222-3p** miR-454-3p miR-345-5p miR-25-5p miR-101-3p miR-135a-5p	**miR-181** **miR-21 (h; d)** miR-375 **miR-34a (d)** **miR-146a** miR-23b **miR-29**	miR-143-3p miR-223-3p miR-410-3p miR-324-5p **miR-155 (d)** miR-1-3p
	**Decreased**	miR-579 miR-574-5p miR-570-3p miR-378e miR-302d-3p **miR-16-5p**	miR-495-3p miR-23a-3p miR-23b-3p miR-149-5p miR-191-5p	miR-154 miR-490-5p **miR-630** miR-639 miR-675b
**Obesity**	**Elevated**	miR-31-5p miR-2355-5p miR-122 **(h; d)** miR-143 **(h; d)** miR-130b miR-34a (*, d)	**miR-199a** miR-222 miR-142-3p miR-423-5p miR-486-3p miR-29a	miR-378 miR-486-5p **miR-140-5p** miR-142 miR-146b **miR-27a**
	**Decreased**	miR-206 miR-28-3p	**miR-221** miR-320a	
**Hypertension**	**Elevated**	miR-21 (*; d)	**miR-143 (o; d)**	miR-122 **(o; d)**
	**Decreased**	**miR-145** miR-27a	miR-133a **miR-145-5p**	
**Dyslipidemia**	**Elevated**	miR-34a (*) miR-218 miR-132 miR-143 **(o; h)** miR-21 (*; h)	miR-122 **(o; h)** miR-155 * miR-3129-5p miR-20b	miR-9-5p miR-320d miR-301a-5p **miR-155-5p**
	**Decreased**	MV-miR-21	MV-miR-122	MV-miR-155

MV, microvesicle.

miRNAs that occur in the same expression pattern in any of the listed risk factors and at least one complication from **Table 1B** are bolded. miRNAs that occur in the same expression pattern in at least two of the listed risk factors are marked: *also in diabetes; o, also in obesity; h, also in hypertension; d, also in dyslipidemia.

**Table 1B:** The miRNAs described in the article and their expression in individual complications.

Complication	Expression	miRNA		
**Endothelial dysfunction**	**Elevated**	miR-222-3p (*) miR-125a-5p	miR-342-3p miR-365b-3p	miR-34a (*; d) miR-126-5p
	**Decreased**	miR-5100 miR-451a	miR-630 (*) miR-16-5p (*)	miR-200c-3p
**Carotid intima-media thickness**	**Elevated**	miR-675-3p	miR-29b	miR-146a (*)
	**Decreased**	miR-218-5p	miR-199a-3p	miR-532-5p
**Arterial stiffness**	**Elevated**	miR-92a miR-21 (*; h; d)	miR-34a-5p miR-122-5p	
	**Decreased**	miR-190a-5p miR-375-3p	miR-454-3p **miR-145 (h)**	
**Atherosclerosis**	**Elevated**	miR-155 (*; d) **miR-126 (o)** miR-17-5p miR-216a miR-148a-3p miR-146a-5p	miR-200b miR-33a/b miR-230d miR-214-3p miR-126-3p	**miR-140-5p (o)** miR-602 miR-335-5p miR-30 miR-361-5p
	**Decreased**	miR-98 miR-31 **miR-221 (o)** miR-17-3p miR-141 miR-222 miR-21	miR-210-3p miR-378a miR-103-3p **miR-145-5p (h)** miR-21-3p miR-204	miR-590-3p miR-34a miR-217 miR-20a miR-486-5p miR-301a-5p
**Diabetic cardiomyopathy**	**Elevated**	miR-155 (*; d)	miR-200c	
	**Decreased**	miR-30c	miR-181a	
**Nephropathy**	**Elevated**	miR-377 miR-146a (*) miR-29 (*)	miR-192 miR-200	miR-195 **miR-155-5p (d)**
	**Decreased**	miR-93		
**Retinopathy**	**Elevated**	miR-23a miR-200b-3p miR-200 (advanced stage)	miR-146a (*) (advanced stage) miR-21-3p	miR-30b-5p miR-155 (*; d)
	**Decreased**	miR-17-5p miR-20a-5p miR-93	miR-106 miR-150-5p	miR-200 (early stage)
**Neuropathy**	**Elevated**	**miR-27a (o)**	**miR-199a (o)**	miR-499a
	**Decreased**	**miR-146a** miR-155-5p	miR-25 miR-302	

miRNAs that occur in the same expression pattern in any of the risk factors listed in **Table 1A** (*diabetes; o, obesity; h, hypertension; d, dyslipidemia) and at least one complication are bolded.

## 2 Type 1 diabetes as a cause of cardiovascular complications in pediatric population

Type 1 diabetes is undoubtedly a serious factor influencing the occurrence and development of cardiovascular complications in children and adolescents (22). The most unfavorable factors influencing diabetes complications are early age of onset and long duration of the disease. For patients diagnosed before the age of 10, life expectancy was reduced by almost 18 years for women and about 14 years for men (20). The most important cardiovascular complications of childhood diabetes include ED, increased cIMT, aortic stiffness, and progressive atherosclerosis, which may lead to an increased risk of myocardial infarction or stroke in early adulthood (6). Also worth mentioning are, diabetic cardiomyopathy, and microvascular complications such as retinopathy, neuropathy and nephropathy.

In recent years, many miRNAs have been studied for the prognosis of childhood T1D. Among the studied miRNAs that would play a key role in accurate prediction of diabetes, miR-146a was highlighted as the most important, followed by miR-29, along with miR-17, miR-20, miR-24, miR-126, miR-133, miR-210, miR-223, miR-320, miR-375 (23, 24). Moreover, significantly increased levels of miR-144-5p, miR-222-3p, miR-454-3p and miR-345-5p were found in sera of children with recently diagnosed T1D compared to the non-diabetic control group (25). In relation to long-term disease in children, miR-25-5p was shown to be increased, while miR-579, miR-574-5p, miR-570-3p, miR-378e, miR-302d-3p and miR-16-5p were decreased in these patients (26). Six more miRNAs were identified with differential levels between young people with long-term illness and healthy controls; miR-101-3p, miR-135a-5p, miR-143-3p, miR-223-3p, and miR-410-3p were upregulated, and miR-495-3p were downregulated (27). In direct comparison with healthy peers, miR-181 overexpression was also found in T1D patients, and due to its negative correlation with C-peptide levels, studies indicate that it may play a significant role in pancreatic β-cell dysfunction (28). Another study indicated that doubling of miR-197-3p at 3 months was the strongest predictor of residual beta-cell function 12 months after diagnosis in children with T1D and corresponded to a six-fold higher stimulated C-peptide level (29). Moreover, doubling of miR-24-3p and miR-146a-5p at 3 months corresponded to a 4.2% and 3.5% lower insulin-corrected HbA1c level at 12 months, respectively. It has been proven that the preservation of C-peptide may play a substantial role in delaying the occurrence of vascular complications in type 1 diabetes (30), therefore, the miRNAs discussed above may be of great importance for analyzing the possibility of maintaining residual β-cell secretion in long-term disease and thus for preventing its complications. However, it is still unknown which panel of miRNAs may have a protective effect in chronic childhood diabetes, or which miRNA expression may negatively influence the acceleration of the development of cardiovascular diseases in later life.

β-cells in the pathogenesis of type 1 diabetes are continuously exposed to inflammatory cytokines influencing their apoptosis. After exposure to interleukin-1b and interferon-γ, miRNA expression assessment showed increased levels of miR-21, miR-23b, miR-34a, and miR-146a, whereas miR-23a-3p, miR-23b-3p, and miR-149-5p were decreased (31). Other studies suggest that increased expression of miR-155 in immune cells may also contribute to β-cell death in type 1 diabetes (8). Studies indicate that miR-21 influences macrophage metabolism, resulting in a shift towards oxidative phosphorylation in naive and inhibition of glycolysis in pro-inflammatory macrophages, which plays a key role in the inflammatory response mediated by them (19). Circulating plasma miR-1-3p and miR-16-5p have also been found to directly influence genes associated with cardiovascular pathologies, such as GOSR2, ANK3, MTMR3 (32) and thus, in combination with glycemic control, may potentially serve as prognostic biomarkers to help prevent the development of these particular complications. Bellini et al. found reduced levels of miR-191-5p in diabetic patients compared with controls, as well as an inverse correlation between the levels of this molecule and chronic vascular complications of diabetes (33).

Hyperglycemia itself, which is both a symptom and a consequence of diabetes, induces a series of pathological changes in small vessels, arteries and peripheral nerves and causes tissue damage in endothelial cells by increasing reactive oxygen species. Moreover, hyperglycemia-induced oxidative stress promotes LDL oxidation, which is associated with increased cIMT in youths with T1D and poor glycemic control (34), and promotes atherosclerosis in several ways. In the study by Erener et al., HbA1c levels significantly negatively correlated with miR-324-5p, which was present in high levels in the serum of children with newly diagnosed type 1 diabetes, while, interestingly, they showed a positive correlation with miR-154, miR-490-5p, miR-630, miR-639, miR-675b, which expression in serum was low (25). It can therefore be suspected that these miRNAs may be released from endothelial cells in a hyperglycemia-induced process, reflecting early tissue damage.

As can be seen, miRNAs play a key role in pancreatic β-cell function, regulating their response to immunological, inflammatory, and metabolic factors. This highlights the importance of miRNAs as potential biomarkers in the diagnosis, prediction, and progression of diabetes complications, as well as therapeutic targets.

## 3 Risk factors for early vascular changes in childhood type 1 diabetes and future cardiovascular complications

Not only diabetes itself, but also comorbidities such as obesity, hypertension or dyslipidemia, the incidence of which is much higher in children with T1D than in their healthy peers, constitute additional risk factors provoking the development of later cardiovascular complications. In this paragraph, we would like to focus on the miRNA alterations that occur in the above-mentioned conditions and may influence early vascular changes that can become apparent already at a young age, ultimately leading to cardiovascular diseases in patients with diabetes.

### 3.1 miRNAs in obesity and obesity-mediated inflammation

Obesity has become a growing problem among children, especially those with type 1 diabetes, and is a documented risk factor for many diseases, including metabolic and cardiovascular disorders (35). Both diabetes and obesity alone are undisputed threats for cardiovascular health of the population, consequently the overlap of the two above conditions magnified the likelihood of complications (36). A multicenter, longitudinal study found that a large proportion of young people with type 1 diabetes who were overweight or obese during adolescence had increased body weight also in adulthood (37). Research suggests that, as in adulthood, elevated body mass index (BMI) in childhood and adolescence appears to be a key factor in higher cardiovascular risk in later life (38, 39). In adolescents and young adults with diabetes, central obesity may contribute to the development of hypertension (40). In the DCCT, excessive weight gain was associated not only with increased blood pressure but also with dyslipidemia and, in the follow-up study, with more extensive atherosclerosis (41). Furthermore, in adolescents with type 1 diabetes, the only modifiable cardiovascular risk factor that predicted cIMT was BMI z-score (42).

The regulation of adipocyte differentiation, lipid metabolism, and energy homeostasis has been shown to be mediated by miRNAs. Furthermore, excessive nutrition can alter the expression of miRNAs in adipocytes, leading to abnormal adipose tissue function (43). Studies show that circulating miRNAs are differentially expressed in obese/overweight children compared to their normal weight peers. One study found a two-fold augmentation in miR-31-5p, a three-fold augmentation in miR-2355-5p, and a 0.5-fold decline in miR-206 expressions in corpulent children (44). A significant increase in the concentration of miR-122, miR-143 and miR-199a was also observed in obese children compared to the control group (45). Additionally, miR-200c-3p, miR-190a, and miR-95 showed differential regulation in obese preschool children with insulin resistance both in the fasting state and 2 hours after an oral glucose tolerance test, potentially acting as biomarkers of insulin resistance in this group (46). The results presented in the review by Oses et al. unveil that miR-222, miR-142-3p, and miR-140-5p are significantly overexpressed in children and adolescents with obesity (47). Moreover, decreased levels of miR-28-3p and miR-221 as well as increased levels of miR-130b, miR-142-3p, miR-423-5p, miR-486-3p and miR-486-5p may prove to be novel biomarkers for estimating the risk of childhood obesity. Both miR-142-3p and miR-486-5p inhibit the expression of the transcription factor FoxO1 (48), which happens to be one of the main mediators of triglyceride metabolism and/or insulin action (49). Elevated plasma miR-486 and miR-142 levels in obese children may be involved in the development of obesity complications such as subclinical inflammation, insulin resistance, and hypertension in later life. Reduced levels of circulating miR-28-3p and miR-221 have been detected in obese children, which seem to be responsible for compensatory mechanisms that reduce the metabolic aberrations of obesity. It has been shown that miR-28-3p is negatively correlated with adiponectin levels, therefore its lower expression may to some extent induce the production of this antidiabetic adipokine, which may have a positive effect not only on insulin sensitivity in prepubertal age but also ultimately protect against diabetes in adulthood (48). In the case of miR-221, its decreased levels were also found in adults with morbid obesity, whereas its increased expression was shown after surgical intervention (50).

In addition, studies indicate that obesity may play a role in increasing the secretion of proinflammatory cytokines, causing chronic low-grade inflammation, as well as the potential role of miRNAs in this process. In a study conducted on a pediatric population, compared to the control group, obese children had increased levels of L - 6, hsCRP, and TNF-α, which positively correlated with levels of miR-29a and miR-122 (51). miR-378 as well as miR-146b may prove to be potential novel targets in controlling inflammation in adipose tissue, as increased expression of both was observed in response to IL - 6 and TNF-α in mature human adipocytes (52, 53). Similarly, in mice studies, TNF-α, the level of which increases already at a very early stage of obesity, upregulates miR-34a, and furthermore, miR-34a expression in visceral fat gradually rises with the development of obesity, while its selective deletion protects against the exacerbation of meta-inflammation induced by dietary stress (54). An increase in miR-27a levels was also observed along with aggravated expression of pro-inflammatory cytokines, macrophage influx and M1 macrophage polarization (55).

### 3.2 miRNAs in hypertension

Children with type 1 diabetes are more susceptible to hypertension than their healthy peers. Higher blood pressure can consequently lead to many microvascular complications and is also a major risk factor for serious macrovascular diseases, including cardiovascular diseases. There are many studies on hypertension and its associated miRNAs, although few studies specifically focus on the pediatric population. A study conducted on 22 children with hypertension showed that increased expression of miR-145 after exercise negatively correlated with both systolic and diastolic blood pressure (56). On the other hand, experimental work in rats suggests an unfavorable role of miR-145 in the development of systemic hypertension and may be involved in epigenetic changes influencing hypertension-induced organ damage (57). In adult patients with hypertension, miR-21 levels have been shown to be elevated and positively correlated with systolic and diastolic blood pressure, CRP levels, and parameters reflecting asymptomatic organ damage, such as microalbuminuria and cIMT (58), which may contribute to its potential as a biomarker heralding this damage without prior clinical manifestation. In another study, Nemecz et al. found a negative correlation between diastolic blood pressure and both plasma miR-143 and miR-122 concentrations (59). Moreover, serum concentrations of miR-27a and miR-133a were lower in individuals with newly diagnosed hypertension compared with normotensive individuals, making them potential biomarkers for predicting or preventing hypertension (60). In the study by Barruta et al. conducted on a large group of individuals with long-term type 1 diabetes, the level of miR-145-5p in serum was significantly lower in hypertensive patients than in normotensive patients and inversely correlated with hypertension, regardless of age, sex and duration of diabetes (61). Nevertheless, more research is needed to determine whether the above considerations are confirmed in the pediatric T1D population.

### 3.3 miRNAs in dyslipidemia

Dyslipidemia is an immense problem in children and adolescents with T1D. A recent study showed that the prevalence of dyslipidemia was 67.3%, with HbA1c being the most important modifiable factor determining its occurrence (62).

Both small and large blood vessels can be affected by lipid imbalances, increasing the risk of atherosclerotic plaque formation and subsequent cardiovascular disease. miR-34a expression was higher in patients with type 1 diabetes and dyslipidemia compared with those without dyslipidemia and showed a strong independent association with total cholesterol and low-density lipoprotein cholesterol (63). Overexpression of miR-34a is thought to be associated with the severity of several dyslipidemia-related disorders, e.g., nonalcoholic fatty liver disease or coronary artery disease (64, 65). In addition, the expression of miR-218, miR-132, and miR-143 was found to be significantly increased in both circulating microvesicles (MV) and plasma of diabetic patients with dyslipidemia (59). Furthermore, three more miRNAs in MV were downregulated: miR-21, miR-122, and miR-155, while the same plasma miRNAs were increased in diabetic dyslipidemia. Moreover, MV-miR-21 positively correlated with plasma cholesterol levels, MV-miR-122 negatively correlated with LDL-C levels, and MV-miR-155 positively correlated with cholesterol levels and negatively with HDL-C levels (59). Macrophage cholesterol efflux capacity has been identified as a predictor of cardiovascular diseases. Interestingly, changes in this process in adolescents are not associated with BMI (occurring in both obese and non-obese youths), inflammation or insulin resistance and occur before the main changes in lipid profiles, and may be partially driven by adipocyte-derived EV miRNAs, in particular miR-3129-5p miR-20b, miR-9-5p, miR-320d, miR-301a-5p, miR-155-5p (66).

## 4 Early preclinical vascular changes in childhood type 1 diabetes

### 4.1 miRNAs in endothelial dysfunction

**Figure 2:**
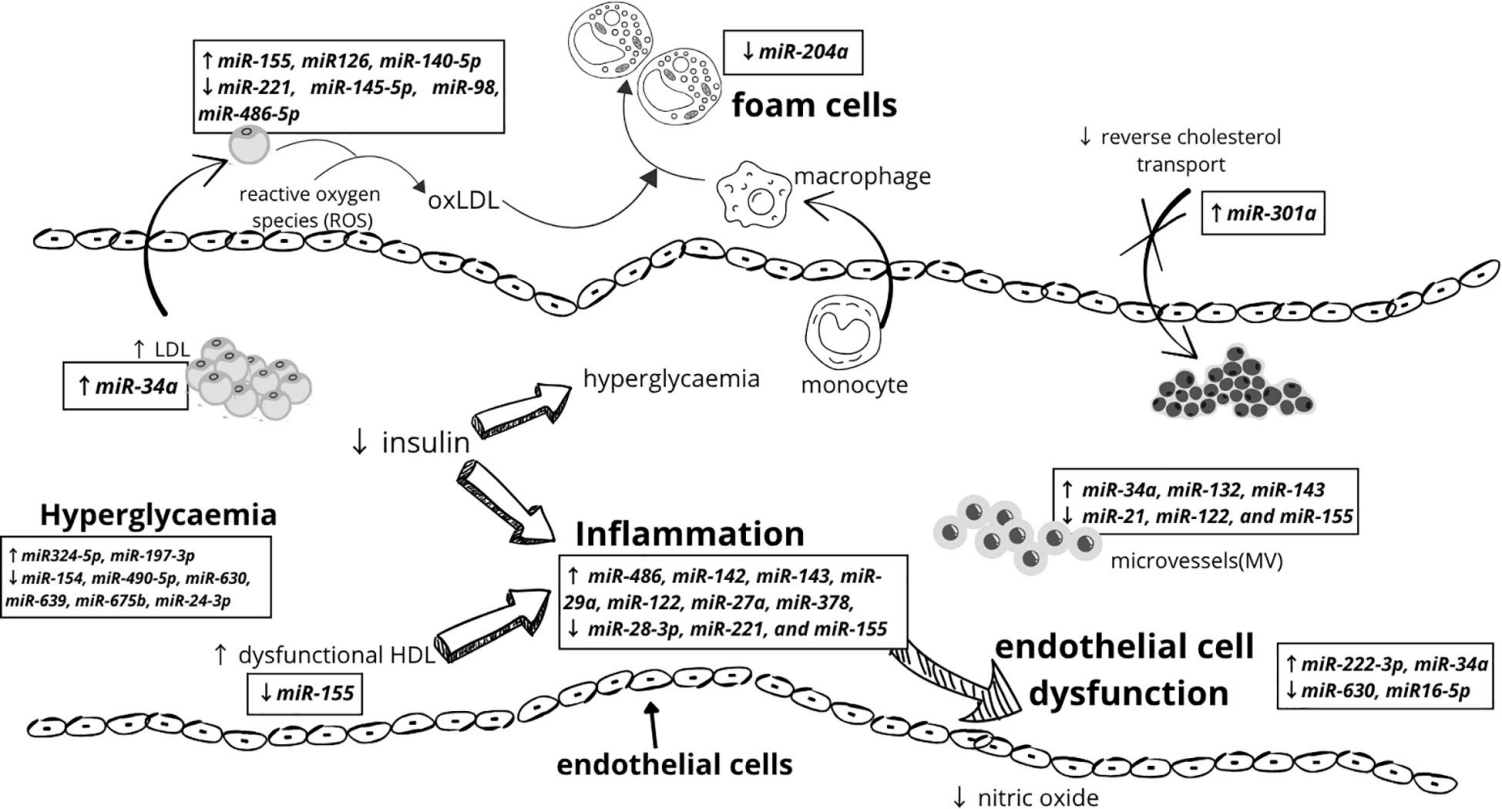
Mechanisms underlying cardiovascular diseases early pathogenesis in children with type 1 diabetes and miRNAs involved in the process.

Endothelial dysfunction (ED) refers to the loss of normal homeostatic function of blood vessels and is characterized by altered vasodilatory/constrictive functions and increased inflammatory activity. It is associated with the development of vascular complications such as hypertension, coronary artery disease, and chronic heart failure. The main factors responsible for the pathogenesis of ED in T1D are uncontrolled hyperglycemia, glycemic variability and low endogenous insulin concentration. All of the above lead to the generation of multiple biochemical factors, increased expression of classes of genes associated with inflammation and cellular stress, which disrupts the structural and functional integrity of the endothelium of vascular structures, thereby increasing progressive low-grade inflammation, inhibiting endothelium-dependent relaxation, and causing potential susceptibility to apoptosis (67). Data suggest that endothelial dysfunction and changes in peripheral vascular dynamics may occur as early as 5 years after the onset of T1D (68). Numerous miRNAs have been studied with the potential to play a significant role in regulating endothelial cell function, inflammation, and vascular integrity, making them potential indicators of endothelial health. Increased levels of circulating miR-222-3p have been shown to cause endothelial dysfunction and were found to be elevated in hyperglycemia (25), which may serve as a useful biomarker to monitor the effects of glycemic control on the vascular endothelium. Khalyfa et al. identified three miRNAs previously associated with cardiomyopathy, namely miR-125a-5p, miR-342-3p, and miR-365b-3p, as potential biomarkers encompassing three biological pathways that may have pathophysiological relevance to abnormal endothelial function in children (69). The researchers also found that in children with ED, the levels of miR-5100, miR-451a, miR-630, and the previously mentioned miR-16-5p were reduced, while after effective treatment there was a significant increase in the levels of the latter two miRNAs to the levels found in healthy individuals. Conversely, transfection of exosomes from individuals without evidence of endothelial dysfunction with a miR-630 inhibitor provoked a functional phenotype of endothelial dysfunction. Gene target discovery experiments conducted by this research group additionally demonstrated that miR-630 regulates as many as 416 gene targets in endothelial cells, including nuclear factor erythroid 2-related factor 2 (Nrf2), AMP-kinase, and tight junction pathways (70). Other miRNA, videlicet miR-34a, showed higher levels in children with T1D compared to their healthy peers in relation to serum endoglin and intercellular adhesion molecules (ICAM) (63), which are known to be involved in, among others, angiogenesis, vascular remodeling and endothelial cell activation (**Figure 2**).

Bakhashab et al. concluded that miR-200c-3p may be an early marker of subclinical ED in T1D patients, finding that it was significantly reduced in peripheral blood mononuclear cells of these patients and negatively correlated with HbA1c, interleukin-7, vascular endothelial growth factor-C, and soluble vascular cell adhesion molecule-1, whereas positively correlated with many markers of vascular health: circulating endothelial progenitor cells as well as proangiogenic cells (71). Furthermore, overexpression of miR-200c in nondiabetic endothelial cells leads to reduced endothelium-dependent relaxation, and inversion of this effect was achieved with anti-miR-200c in diabetic mice, thus postulating that it could be a potential target for intervention in vascular diseases (72). The last miRNA we want to discuss that has been shown to have proangiogenic and cardioprotective effects is miR-126-5p. Its lower expression has been shown to be associated with better vascular health, whereas its upregulation, likely enhanced by hyperglycemia and inflammation in T1D, may be a compensatory response (73).

### 4.2 miRNAs in carotid intima-media thickness

CIMT, i.e. the thickness of the common carotid arteries, is considered an important indicator of vascular complications in type 1 diabetes, which is measured primarily by ultrasound (74). Studies show that this marker is often significantly elevated in children with diabetes due to the pathogenesis of long-term hyperglycemia compared to healthy people of a similar age, and the highest results occur in the group with pre-existing vascular complications and in children with obesity (6). It has also been shown that the occurrence of insulin resistance and high fasting glycemia intensifies the increase in cIMT (74). Additionally, boys show higher cIMT values ​​compared to girls, especially in late adolescence. Increased cIMT is associated with an increased risk of stroke and heart attack already in young adulthood (75). Studies have shown a strong correlation of cIMT with the expression of circulating miRNA, due to increased activation of the pathways regulating the activity of vascular endothelium and reduced regulation of its apoptosis. According to studies, the occurrence of miR-675-3p currently correlates most with atherosclerotic loss (76). MiR-29b and miR-146a may also prove to be potentially useful in diagnostics (75). On the other hand, miR-218-5p and miR-199a-3p (77) negatively correlate with cIMT (78) and miR-532-5p (79) demonstrated even in the asymptomatic phase of atherosclerosis. These miRNAs might serve as potential biomarkers for detecting early stages of atherosclerosis before clinical symptoms appear.

### 4.3 miRNAs in arterial stiffness

Young patients with type 1 diabetes have been found to have increased arterial stiffness compared to healthy individuals (80), which is considered a prognostic factor for developing atherosclerosis and an independent predictor of all-cause and cardiovascular mortality in later life. The primary effect of arterial stiffening is a loss of compliance and buffering potential, which results in increased pulse wave propagation and accelerates tissue damage in organs with numerous low-resistance capillaries, namely the kidneys, heart or brain, because they are more sensitive to pressure oscillations and more susceptible to damage from excessive, unbuffered pulsation of large arteries (81). Commonly used non-invasive methods of assessing arterial stiffness, including pulse wave velocity (PWV) and augmentation index (AIx), have been reported to predict future cardiovascular events. Importantly, a significant increase in PWV of 0.145 m/s/year over only a 5-year period was observed in adolescents with type 1 diabetes (82). Although the exact mechanism of early arterial remodeling toward stiffer walls remains unclear, additional information suggests an association between PWV in T1D and the additional cardiovascular risk factors mentioned earlier. Less commonly used magnetic resonance imaging (MRI) techniques can directly assess central aortic stiffness by measuring hemodynamic parameters. Of possible clinical importance is the growing evidence suggesting that MRI-based central aortic stiffness may be reversible in response to medical intervention or exercise (83, 84), hence it is worthwhile to explore new methods that can reverse these vascular changes. It has been speculated that circulating levels of miR-92a may be an important biomarker and potential therapeutic target for both hypertension and arterial stiffness. In an adult study, extracellular endothelium-derived miR-92a, which levels were positively correlated with PWV, may play an active role in promoting arterial stiffness by regulating the communication between endothelial cells and vascular smooth muscle cells. Furthermore, exogenously administered to mice locked nucleic acid-modified antisense miR-92a ameliorated angiotensin II-induced hypertension and arterial stiffness (85). In a study evaluating children with hypertension, an increase in miR-145 expression following physical exercise was described, which negatively correlated with 24-h peripheral PWV (56). Recently, in adults, reduced miR-21 expression was observed to lead to lower arterial stiffness in patients with hypertension at baseline as well as after antihypertensive treatment, regardless of blood pressure (86). Both studies offer hope for enabling the use of miRNA as a potential prognostic marker and future therapeutic target. In individuals over 50 years of age, higher expression of miR-190a-5p, miR-375-3p, and miR-454-3p was associated with lower PWV, whereas miR-34a-5p and miR-122-5p were associated with higher PWV (87).

## 5 Threatening future cardiovascular complications in type 1 diabetes

Various studies confirm that the incidence of cardiovascular risk factors is elevated among children and adolescents with diabetes, and in many cases these factors are already present at the time of diagnosis (88). Diabetes escalates the risk of cardiovascular disease, premature cardiovascular mortality, by at least twofold, and all-cause mortality by fourfold in young people. Furthermore, cardiovascular outcomes and mortality are inversely correlated with age at onset of diabetes; suggesting that the earlier the onset, the greater the risk (20). The additional comorbidity of obesity increases the risk of both disease-specific and all-cause mortality (89). In this chapter, we will discuss and attempt to summarize recent findings on cardiovascular complications of diabetes that seem to be regulated by miRNAs.

### 5.1 miRNAs in atherosclerosis

Atherosclerosis is a pathological process characterized by the accumulation of atherosclerotic plaques within the arterial walls, leading to their progressive hardening and luminal narrowing. This process may commence significantly earlier than commonly assumed - often during childhood or even in fetal development (90). While severe cardiovascular complications, such as CAD, manifest later in life, the underlying pathogenic mechanisms are initiated at an early age (91).

Numerous miRNAs regulate metabolic pathways associated with plaque formation. Thus, early identification of these biomarkers may help prevent adverse consequences, including myocardial infarction (90), which have been documented already in the second decade of life (92). In newborns and children in the first decade of life, the early stages of atherosclerosis are characterized by the presence of fatty streaks (93). These changes have even been demonstrated in the aorta and coronary arteries (94). As a consequence, this leads to vascular stiffening and accelerated vascular aging (92, 93). Significantly, the presence of fatty streaks is often observed despite normal blood pressure, BMI and cholesterol levels (95).

The initial phase of atherosclerosis involves the accumulation of oxidized low-density lipoprotein (oxLDL) within the subendothelial layer, regulated by miR-155 and miR-148a-3p, miR-146a-5p (96). The effect of miR-155 is expression-dependent, with low expression exerting an anti-atherosclerotic effect (97–99). Conversely, miR-98 inhibition enhances the secretion of chemokines and adhesion molecules in human umbilical vein endothelial cells (HUVECs) by suppressing HMGB1 expression (100). These processes are counteracted by miR-31, which regulates E-selectin expression, and miR-221, miR-17-3p, miR-141, and miR-222, which inhibit ICAM - 1 expression, collectively reducing leukocyte adhesion (96, 101).

A particularly intriguing biomarker is miR-126, which exerts dual effects: it offers protective benefits by inhibiting VCAM - 1 expression, yet excessive expression may promote angiogenesis and plaque instability (17, 96, 97).

Following this stage, unstable plaque formation occurs. Several miRNAs contribute to this process, including miR-17-5p, which modulates macrophage proliferation and apoptosis (17, 96); miR-155, which activates TNF-alfa and NLRP3 inflammasomes, exacerbating inflammation and plaque destabilization (101); and miR-216a, which promotes M1 macrophage polarization by activating telomerase in the Smad3/NF-κB pathway (102). Additionally, miR-200b is activated under hypoxic conditions, leading to eNOS destabilization, reduced nitric oxide (NO) levels, and vasoconstriction (97). Furthermore, miR-21 deficiency increases apoptosis and necrosis within the atherosclerotic plaque (102).

Conversely, miR-210-3p exerts protective effects by inhibiting lipid accumulation and inflammation through IGF2 suppression. miR-378a also regulates macrophage phagocytosis via the CD47-SIRPα pathway (102). Lipid metabolism dysregulation significantly contributes to atherosclerosis progression. miR-33a/b enhances lipid accumulation, impairs apoptotic cell clearance, and reduces cholesterol uptake by downregulating CYP7A1 and CYP8B1 (96, 102). Similarly, miR-230d promotes lipid accumulation within the vascular walls. In contrast, miR-103-3p protects endothelial cells via the Notch1 pathway (103); miR-145-5p supports vascular smooth muscle cells (VSMCs) by reducing their proliferation and migration (96). miR-21-3p – also stimulates VSMC (104); and miR-204 inhibits foam cell formation by reducing SR-A and CD36 expression (104, 105).

Oxidative stress, driven by excessive reactive oxygen species (ROS) production, also plays a crucial role in atherogenesis. For instance, miR-214-3p inhibits GPX4 activity, miR-140-5p and miR-602 influence Nrf2 and SIRT2 expression, while miR-335-5p downregulates SIRT7, all contributing to disease progression (106). Additionally, excessive miR-30 expression has been associated with carotid atherosclerosis in hypertensive patients (17). MiR-20a and miR-486-5p shield endothelial cells from oxLDL-induced apoptosis, while miR-301a-5p modulates ABCA1/ABCG1 transporter expression, facilitating cholesterol efflux from macrophages (106). Notably, specific miRNAs have been linked to distinct pathological conditions. For example, miR-126-3p has been frequently associated with albuminuria (107), whereas elevated miR-361-5p levels have been observed in patients with acute myocardial infarction (17), including young individuals in their second decade of life, among whom substantial atherosclerotic lesions had already been identified during the first decade of life (94).

### 5.2 miRNAs in diabetic cardiomyopathy

Diabetic cardiomyopathy (DCM) is a condition that refers to the structural and functional changes of the heart muscle associated with diabetes, including ventricular hypertrophy, myocardial fibrosis, and reduced heart function, and even heart failure. Type 1 diabetes causes initially impaired diastolic function, then subclinical systolic function with preserved ejection fraction (EF), progressing to heart failure with reduced EF. Children and adults with type 1 diabetes exhibit early signs of cardiomyopathy despite normal EF, because it is a crude marker of ventricular function that declines only late in the disease process. EF may be maintained by myocardial remodeling, despite the presence of significant reductions in longitudinal myocardial shortening located in the subendocardium, which is more susceptible to ischemic injury due to coronary microvascular dysfunction, commonly associated with hyperglycemia and insulin resistance (108). Early detection of diastolic dysfunction and subclinical systolic dysfunction is a priority, as both early pathologies strongly predict poor clinical outcomes (109). DCM occurrence is independent of other risk factors, such as hypertension or coronary heart disease, which is why the search for biomarkers for DCM prediction, prevention or treatment absorbs a lot of scientific efforts, including the search for appropriate miRNAs.

*In vitro* studies have shown that hyperglycemia increases miR-155 expression in cardiomyoblasts, leading to increased expression of profibrotic genes (e.g.: NRF2/HO-1 signaling pathway) and mitochondrial dysfunction. Furthermore, a miR-155 inhibitor can attenuate cardiac fibrosis, making miR-155 a promising target for the prevention or treatment of DCM (110). Moreover, diabetic heart cells showed significantly reduced expression of miR-181a and miR-30c and increased expression of their target genes p53 and p21, which are key regulators of cardiomyocyte hypertrophy, mediating the pro-hypertrophic effects of hyperglycemia (111). Furthermore, increased expression of miR-200c was observed in the diabetic cardiomyopathy model and in exposed patients cardiomyocytes to high glucose levels, while subsequent reversal of cardiomyocyte hypertrophy was achieved by inhibition of miR-200c (112).

DCM is an urgent problem because changes in the heart are a significant factor influencing mortality among the diabetic population, hence the need to continue research on its indicators in the earlier stages of type 1 diabetes in children, as such studies are unfortunately scarce.

## 6 Microvascular complications in type 1 diabetes

Statistics on vascular complications in type 1 diabetes in children and adolescents may vary depending on the study group. According to one study, in a group of 155 individuals with a mean age of 14.4 ± 2.1 years, the incidence of microangiopathy was 16.1% (113); 50% of patients developed complications within 5 years of diagnosis of type 1 diabetes, and 25% within 2 years (114). Effective diabetes treatment has significantly reduced the number of patients with microvascular complications in recent years. The percentage of patients with diabetic retinopathy has decreased from 45% to 5%, with neuropathy from 50% to 15%, and with nephropathy from 25% to 4 - 9%, depending on the study group (115).

### 6.1 miRNAs in nephropathy

Diabetic nephropathy (DN) is characterized by pathological changes including thickening of the glomerular and tubular basement membranes, mesangial matrix expansion, and eventual progression to glomerulosclerosis and tubulointerstitial fibrosis (61). DN is a leading cause of end-stage renal disease (116, 117). Evidence suggests that structural glomerular alterations often precede the clinical manifestation of microalbuminuria and abnormal albumin-to-creatinine ratio (ACR) values in adolescents often precede the clinical onset of microalbuminuria. A correlation has been shown between ACR and markers of glomerular hyperfiltration. Higher ACR activity in adolescents has been associated with the future development of advanced stages of diabetic nephropathy and cardiovascular disease. This is confirmed by the marker hs-CRP (high sensitivity C-reactive protein; a factor measuring the risk of cardiovascular diseases), which increases in parallel with the increase in ACR level (118).

Recent studies have identified significant alterations in the urinary levels of miRNAs such as elevated level of miR-377 and reduced level of miR-216a in pediatric patients with type 1 diabetes mellitus (T1DM). miR-377 has been implicated in mesangial cell dysfunction and oxygenase-1 (HO - 1), a critical antioxidant enzyme, exacerbating podocyte injury and albuminuria (117).

miRNAs such as miR-29, miR-192, and miR-200 regulate extracellular matrix (ECM) synthesis, with reduced expression of miR-29 in DN leading to increased collagen production and ECM accumulation (119, 120). It operates through the mechanism of renal fibrosis. Similar effects have been included by increased expression of miR-146a (9). miRNAs also regulate other pathogenic processes in DN, including oxidative stress (miR-377) and podocyte apoptosis (miR-195). Furthermore, reduced expression of miR-93 in podocytes and endothelial cells increases vascular endothelial growth factor (VEGF) expression, enhancing glomerular permeability and albuminuria, which are characteristic features of diabetic nephropathy (117). Addictionaly, miR-155-5p, exhibits increased expression in hyperglycemia. This leads to irreversible fibrotic changes in the renal tubular epithelium. The expression of miR-155-5p has also been observed in glomerular mesangial cells (121).

### 6.2 miRNAs in retinopathy

Microvascular dysfunction, characterized by altered interactions between endothelial cells and pericytes, is a key event in the pathogenesis of diabetic retinopathy. According to a study conducted between 2000 and 2020 on approximately 150,000 young people, its prevalence is 5.8% (122), while for severe retinopathy it is less than 1%. EVs derived from mesenchymal stem cells cultures can enter pericytes, causing their detachment, migration, and stimulating angiogenesis. Studies have shown that the miRNA profiles in EVs from diabetic retinopathy patients differ significantly from those of healthy controls and non-complicated patients with diabetes (123, 124).

It has been shown that lowering the level of miR-17-5p, miR-20a-5p (125), miR-93 and miR-106 causes retinal angiogenesis and thus the development of retinopathy already in premature infants (126). The results of another study showed that the expression of miR-23a and miR-200b-3p was significantly elevated in patients with retinopathy (126). Notably, miR-21-3p, miR-30b-5p, and miR-150-5p play a crucial role in vessel destabilization and angiogenesis. Hypoxia, a characteristic feature of the diabetic eye, activates HIF - 1α (a critical regulator of proangiogenic genes such as VEGF) and associated miRNAs, including miR-21-3p and miR-30b-5p, further contributing to vascular instability and abnormal vessel growth. In contrast, miR-150-5p, which normally inhibits angiogenesis by upregulating ITGA6, is downregulated in patients with DR (124).

It is in different way with miR-200. It has a dynamic expression pattern dependent on the stage of the disease. In the early stages of retinopathy, its reduced expression leads to increased VEGF levels and vascular permeability in infants (126). In advanced stages, miR-200b is overexpressed, contributing to increased oxidative stress by inhibiting Oxr1 (127).

MiR-146a plays a protective role by reducing the activation of inflammatory genes, such as MCP - 1 and ICAM - 1, through negative feedback in the NF-κB pathway. In addition, animal models have shown that overexpression of miR-146a limits pathological changes in the retina, such as overproduction of extracellular matrix proteins (128).

Diabetic retinopathy is currently a rare complication of type 1 diabetes in children. The incidence varies, because it depends on the age, gender and social environment of the population. According to studies conducted in the USA, it is estimated at <4% in children and adolescents with type 1 diabetes. Additionally, maintaining HbA1c <8% significantly reduces the risk of this complication, which confirms how important it is to maintain normal glycemia in the long term (129).

### 6.3 miRNAs in neuropathy

Dysregulation of miRNAs in the central nervous system causes progressive neuronal loss, resulting in neurodegenerative diseases. miR-124 and miR-298 (130) is responsible for the most common one, Alzheimer’s syndrome, while miR-133b affects Parkinson’s disease (131). miRNAs regulate neurogenesis and dendritic growth. Their insufficient concentration contributes to neuronal apoptosis. Moreover, studies indicate their role in maintaining synaptic plasticity (132).

Changes have not been demonstrated in children, however, elevated plasma levels of miR-21 and miR-210, along with decreased miR-126 concentrations, correlate with an increased risk of nervous system diseases in early adulthood (133). In diabetic polyneuropathy (DPN), microR-146a exerts neuroprotective effects by reducing inflammation and improving myelination and preserving nerve structure (128, 134). MiR-146 located in circulating mononuclear cells modulates inflammatory response in diabetic peripheral neuropathy. Additionally, miR-146a (97)and miR-155-5p exhibits a protective role against early cardiovascular autonomic neuropathy (CAN) (121). A protective role is also played by higher concentrations of miR-25 (134), miR-302, which through various metabolic pathways lead to reduced oxidative stress (119). This is unlike miR-27a, miR-499a and miR-199a, which are prothrombotic factors (132, 134). Dysregulated miRNAs may contribute to the development of diabetic foot, which is the most common complication of DPN (135).

## 7 Prevention and interventions

Cardiovascular disease accounts for up to 25 – 50% of deaths in people with type 1 diabetes who have had diabetes for even less than 20 years, and this percentage increases with longer duration of the disease (20). Prevention of cardiovascular events in people with type 1 diabetes is crucial and is based on the promotion of a healthy lifestyle, healthy diet, physical activity, maintaining a healthy body weight, strict glycemic control, maintaining normal blood pressure and LDL-C levels, and monitoring patients during follow-up visits: ACR and examination for neuropathy every year, fundus examination every 1 – 2 years, carotid artery ultrasound recommended every 5 years in the absence of significant preclinical atherosclerosis (136). As most early changes are subclinical, screening for cardiovascular complications is critical to identify adolescents at risk and initiate treatment before irreversible changes occur. The ADA considers the use of age-approved statins, along with nutritional therapy and lifestyle changes, in youth with type 1 diabetes >10 years of age whose LDL-C is ≥130 mg/dL (≥3.4 mmol/L) (4). Current treatment strategies and goals for T1D are based on the results of The Diabetes Control and Complications Trial (DCCT) and its follow-up, the Epidemiology of Diabetes Interventions and Complications (EDIC) trial, which showed that intensive insulin therapy to achieve glycemic control as close to normoglycemia as possible improves cardiovascular risk factors and effectively delays the onset and progression of microvascular and macrovascular complications, with benefits that persist for decades and are associated with reduced all-cause mortality (137). Nevertheless, an association has been demonstrated between glycemic variability, cardiovascular risk factors, future complications, and mortality, regardless of adherence to recommendations for maintaining glycemic targets (138). In addition, hypoglycemia may contribute to cardiovascular complications, including myocardial ischemia and arrhythmias, by causing changes in hemodynamics, coagulation, arterial stiffness, cardiac electrophysiology, and autonomic function (139).

**Figure 3:**
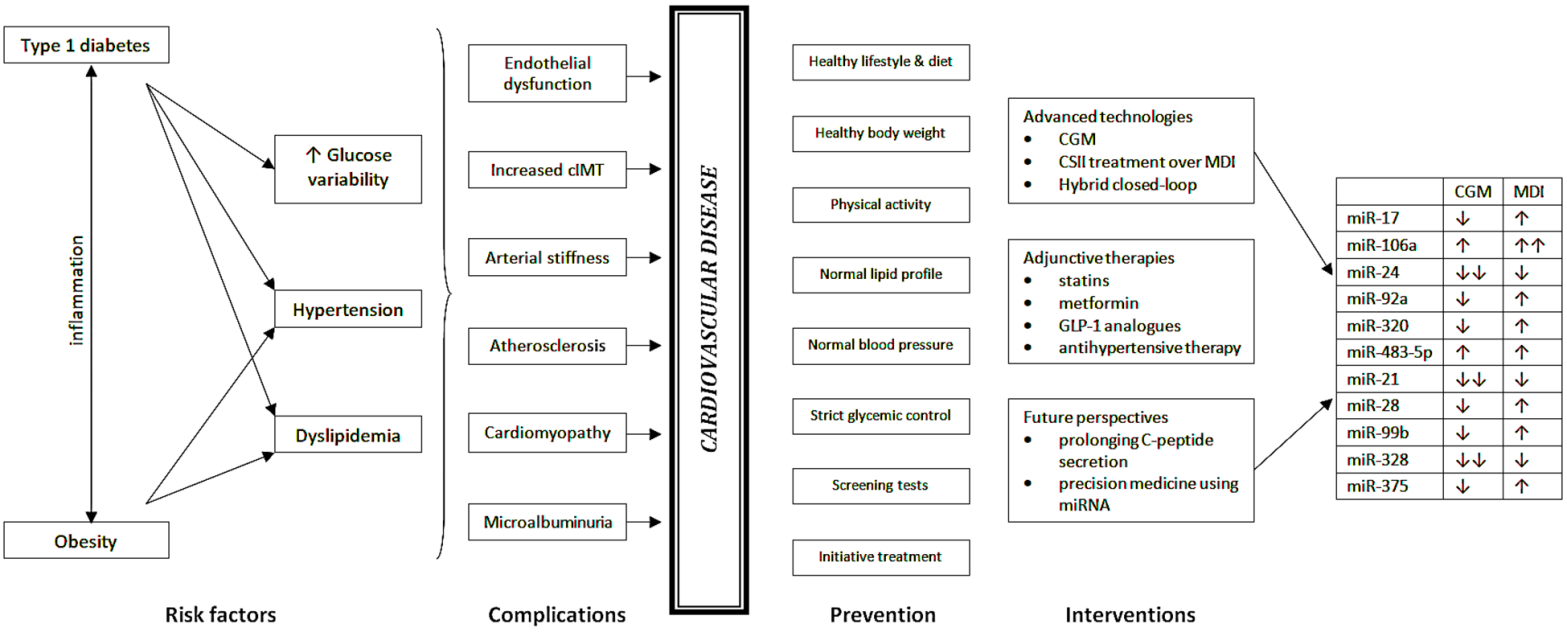
Risk factors and early vascular complications in children with type 1 diabetes predisposing to future CVD as well as possible prevention and interventions strategies in the management of CVD.

Initiation of insulin pump therapy within 6 months of diagnosis of childhood type 1 diabetes was associated with reduced cardiovascular risk, particularly lower mean systolic blood pressure and higher high-density lipoprotein cholesterol (HDL-C), compared with those who initiated CSII within 2 – 3 years of disease onset (140). A multicenter study showed that patients using insulin pumps had increased HDL-C levels and reduced total cholesterol, LDL-C and triglyceride levels, and this effect was maintained after 8 years of follow-up when they also showed fewer cardiovascular events compared to diabetics using MDI (141). Similarly, in a large Swedish registry of 18,168 people with type 1 diabetes, a reduction in coronary heart disease mortality (45%), cardiovascular disease mortality (42%), and all-cause mortality (27%) was observed with pump use compared with MDI use over approximately 7 years of follow-up. It was hypothesized that this may have been due to a reduction in severe hypoglycemia with CSII (142). Interestingly, literature reports that compared to patients using MDI, CSII treatment was associated with lower arterial stiffness independent of other risk factors (143). The use of personal insulin pumps may provide cardiac benefits also by improving endothelial function and overall myocardial performance, as lower cIMT and anteroposterior diameter of the infrarenal abdominal aorta were observed compared to MDI use (144). Systematic use of modern solutions in the treatment of T1D by improving glycemic trends and beyond may positively influence cardiovascular risk factors such as hypertension and dyslipidemia or even protect against the development and progression of macrovascular complications such as circulatory system diseases. Recently, it has been suggested that increased glycemic variability, i.e. the number and amplitude of glycemic excursions measured by CGM as well as HbA1c ​​above target values, may constitute undisputed cardiovascular risk factors (145). A previous study from our institution demonstrated that reducing glycemic variability in a population of young patients with type 1 diabetes using CSII and real-time CGM improves endothelial function by increasing flow-mediated dilation of the brachial artery (146). Current recommendations indicate that hybrid closed-loop systems are the most beneficial treatment option for T1D patients and should be the treatment of choice in the pediatric population since they provide optimal metabolic control while improving the quality of life of both diabetics and their families (147). Furthermore, for patients who have not achieved satisfactory metabolic control with traditional methods of intensive insulin therapy, the use of closed-loop systems may be the only way to improve metabolic control and reduce the risk of acute and chronic complications. A diagram summarizing the risk factors in type 1 diabetes with their subsequent complications leading to cardiovascular diseases, as well as methods of prevention and intervention is presented in **Figure 3**.

In the only study to assess differences in miRNA expression during insulin therapy in children with type 1 diabetes, 27 Australian adolescents without significant differences in baseline characteristics were randomized to CSII or MDI within 3 months of disease onset. There were no significant differences in baseline miRNA expression by treatment, but significantly altered expression of individual miRNAs (measured between baseline and end of follow-up) were noted between the CSII and MDI groups. Expression of miR-17, which has been proven to positively correlate with vascular complications, decreased in patients on CSII and increased in patients on MDI. In addition, miR-106a is seen in higher amounts in diabetics with vascular complications, and its levels increased in patients using both therapies, but to significantly higher levels in patients on MDI therapy. Although decreased expression of miR-24, miR-92a, miR-320, and miR-483-5p has been described in patients with cardiovascular complications, in this study the researchers observed that miR-24 decreased with both treatments, but more so with CSII therapy; miR-92a and miR-320 decreased with CSII and increased with MDI, whereas miR-483-5p increased regardless of treatment method. miR-21 levels decreased to a greater extent during the follow-up in CSII users than in MDI users, whereas increased expression of this miRNA was noted in individuals with nephropathy, retinopathy and high glucose variability. miR-28 and miR-99b, which were shown to predict an increased risk of microalbuminuria, were decreased from baseline in CSII users and increased in the MDI group. miR-328 associated with impaired wound healing in diabetic individuals showed reduced expression compared to baseline in both adolescent groups, but the degree of reduction was less in those using MDI. Compared to baseline, miR-375 levels, associated with beta-cell death, decreased in CSII users and increased in MDI users, but did not correlate with changes in C-peptide. In addition, CSII therapy was associated with lower glycemic variability than MDI therapy (148). Therefore, it can be concluded from this study that early CSII therapy in adolescents with T1D is associated with an altered miRNA expression pattern less adversely related to vascular complications and may be an early sign of better vascular health in adolescents treated with CSII. Nevertheless, replication and further studies as well as long-term follow-up are warranted to validate the data collected here and to explore preventive methods or possible therapeutic targets for preserving or prolonging healthy vasculature.

MiRNAs appear to be promising targets for therapeutic interventions. In obese children and adolescents, miR-320a expression significantly increased after 12 weeks of exercise training and correlated negatively with markers of endothelial dysfunction (149). Increased expression of miR-133a after exercise has been shown to positively correlate with left ventricular mass index, and thus may be a marker of left ventricular hypertrophy in children with primary hypertension (56). Furthermore, it has been suggested that reduced miR-133a expression is associated with the process of cardiac hypertrophy, because this molecule indirectly affects antihypertrophic genes (150), whereas increased miR-133a expression prevents early cardiac fibrosis in diabetes (151). Children with left ventricular hypertrophy may therefore have a greater potential to reverse adverse cardiac changes, and exercise seems to help in this matter. Similarly, obese male adolescents who underwent 6 weeks of exercise combined with a low-calorie diet showed an amelioration in their peripheral vasodilation capacity, indirectly indicating improved vascular endothelial function, which was associated with a reduction in miR-126 expression (152). Together, the data from these studies may indicate a protective effect of exercise, healthy diet and weight loss on endothelial health and reduced cardiovascular risk in children and adolescents as well as demonstrate the involvement of miRNAs in these processes.

## 8 Conclusions

Hitherto, no precise method has been developed to determine the risk of cardiovascular complications in children with type 1 diabetes, and they are usually diagnosed in the advanced stages. Notably, variable miRNAs are important in the pathogenesis, development and course of T1D, additional risk factors which are comorbidities such as obesity, hypertension or dyslipidemia, as well as early vascular changes occurring already in adolescence and later complications in old age. Based on the above considerations, miRNAs do not seem to be only a passive biomarker of developing complications, but may play an active role in the pathogenesis of cardiovascular complications and be of great importance in the development of precision medicine in the future. Knowledge of how the expression of specific miRNAs changes in individual risk factors and then after a specific intervention, identifying and developing anti-miRNAs and miRNA mimetics to inhibit, predict, and treat cardiovascular complications of diabetes in T1D is an active and alluring area of ​​research. Disorder-causing aberrantly upregulated miRNAs can be targeted for silencing or sequestration, whereas those that are downregulated can be transfected to inhibit or cure the disturbances they induce (153, 154). MiRNA may therefore play a tremendous role in precise diagnosis, prevention, delaying cardiovascular complications or determining personalized therapy. Taking into account the above considerations, we hope that miRNA profiling in children with type 1 diabetes will be used in decision-making regarding cardiovascular diseases even in the coming years. However, before miRNA can be widely accepted to use in clinical practice, validation and standardization are necessary, as well as identification of potential factors that may influence false positive or false negative results due to the method of sample collection or storage or technical conditions. It’s important to note that while these findings are promising, they are exceptionally scarce in the discussed pediatric population. We summarize the potential miRNA biomarkers of cardiovascular risk in children with type 1 diabetes in **Table 2**. Further, more detailed analyses on the influence of selected miRNAs on specific risk factors for cardiovascular complications in people with type 1 diabetes, both in developmental and adult age, are necessary.

**Table 2:** Potential miRNA biomarkers of cardiovascular risk in children with type 1 diabetes.

Hyperglycemia	Obesity-mediated inflammation	Hypertension/Arterial stiffness	Dyslipidemia	Endothelial dysfunction
miR-324-5p miR-154 miR-490-5p miR-630 miR-639 miR-675b miR-155	miR-486 miR-142 miR-29a miR-122	miR-145	miR-34a miR-3129-5p miR-20b miR-9-5p miR-320d miR-301a-5p miR-155-5p	miR-125a-5p miR-342-3p miR-365b-3p miR-5100 miR-451a miR-630 miR-16-5p miR-34a miR-222-3p miR-630
